# Comparison of the prevalence and associated factors of hyperactive delirium in advanced cancer patients between inpatient palliative care and palliative home care

**DOI:** 10.1002/cam4.3661

**Published:** 2020-12-12

**Authors:** Jun Hamano, Masanori Mori, Taketoshi Ozawa, Jun Sasaki, Masanori Kawahara, Asumi Nakamura, Kotaro Hashimoto, Kazuhiro Hisajima, Tomoyuki Koga, Keiji Goto, Kazuhiko Fukumoto, Yuri Morimoto, Masahiro Goshima, Go Sekimoto, Mika Baba, Kiyofumi Oya, Ryo Matsunuma, Yukari Azuma, Kengo Imai, Tatsuya Morita, Takuya Shinjo

**Affiliations:** ^1^ Division of Clinical Medicine Faculty of Medicine University of Tsukuba Tsukuba Japan; ^2^ Seirei Mikatahara General Hospital Shizuoka Japan; ^3^ Megumi Zaitaku Clinic Yokohama Japan; ^4^ Yushoukai Medical Corporation Tokyo Japan; ^5^ Soshukai Okabe Clinic Sendai Miyagi Japan; ^6^ Himawari Clinic Chiba Japan; ^7^ Fukushima Home Palliative Care Clinic Fukushima Japan; ^8^ Dr. GON Kamakura Clinic Kanagawa Japan; ^9^ Nozominohana Clinic Chiba Japan; ^10^ Himawari clinic Kumamoto Japan; ^11^ Iwata Home Care Clinic Shizuoka Japan; ^12^ Morimoto Clinic Hyogo Japan; ^13^ Home care clinic Kobe Hyofo Japan; ^14^ Sekimoto Clinic Hyogo Japan; ^15^ Department of Palliative Medicine Suita Tokushukai Hospital Suita Japan; ^16^ Department of Palliative and Supportive Care Aso Iizuka Hospital Fukuoka Japan; ^17^ Department of Palliative Medicine Kobe University Graduate school of Medicine Hyogo Japan; ^18^ Tokyo Metropolitan Cancer and Infectious Diseases Center Komagome Hospital Tokyo Japan; ^19^ Seirei Hospice Seirei Mikatahara General Hospital Shizuoka Japan; ^20^ Department of Palliative and Supportive Care Palliative Care Team, and Seirei Hospice Seirei Mikatahara General Hospital Shizuoka Japan; ^21^ Shinjo‐clinic Hyogo Japan

**Keywords:** advanced cancer patients, hyperactive delirium, inpatient palliative care, palliative home care, place of care

## Abstract

**Background:**

Hyperactive delirium is known to increase family distress and the burden on health care providers. We compared the prevalence and associated factors of agitated delirium in advanced cancer patients between inpatient palliative care and palliative home care on admission and at 3 days before death.

**Methods:**

This was a post hoc exploratory analysis of two multicenter, prospective cohort studies of advanced cancer patients, which were performed at 23 palliative care units (PCUs) between Jan and Dec 2017, and on 45 palliative home care services between July and Dec 2017.

**Results:**

In total, 2998 patients were enrolled and 2829 were analyzed in this study: 1883 patients in PCUs and 947 patients in palliative home care. The prevalence of agitated delirium between PCUs and palliative home care was 5.2% (95% CI: 4.2% ‐ 6.3%) vs. 1.4% (0.7% ‐ 2.3%) on admission (*p* < 0.001) and 7.6% (6.4% ‐ 8.9%) vs. 5.4% (4.0% ‐ 7.0%) 3 days before death (*p* < 0.001). However, multivariate logistic regression analysis revealed that the place of care was not significantly associated with the prevalence of agitated delirium at 3 days before death after adjusting for prognostic factors, physical risk factors, and symptoms.

**Conclusions:**

There was no significant difference in the prevalence of agitated delirium at 3 days before death between inpatient palliative care and palliative home care after adjusting for the patient background, prognostic factors, symptoms, and treatment.

## Graphical abstract

We enrolled 2998 patients in palliative care units and home care. A 169 patients were excluded due to the date of death is unknown. We analyzed 2829 patients: 1882 patients in palliative care units, and 947 patients in home care.

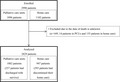

## 1 INTRODUCTION

Delirium is a common but intractable symptom in advanced cancer patients, especially in the palliative care setting.^1^, ^2^, ^3^ Hosie et al. reported that the prevalence of delirium in specialist palliative care inpatient settings varied at the timing of assessment; 13.3%–42.3% at admission, 26%–62% during admission, and increasing to 58.8%–88% in the weeks or hours preceding death.^4^ Previous studies suggested that delirium causes significant distress not only to the patient, but also to the family and health care providers.^1^, ^2^ In addition, delirium makes pain control difficult due to the difficulty in communication.^3^, ^5^, ^6^, ^7^, ^8^ In particular, hyperactive delirium is known to increase family distress and the burden on health care providers.^9^

A recent systematic review reported the prevalence of delirium subtypes; hypoactive, hyperactive, and mixed‐type delirium, at different timings and settings; however, the prevalence of hyperactive delirium in palliative home care is unknown.^10^ This systematic review also suggested that the prevalence of delirium in palliative home care is lower than that in palliative care units (PCUs), but the timing and assessment tools were not standardized.^10^ Mercadante et al. assessed the prevalence of delirium by MDAS in palliative home care and hospice settings on admission and after 1 week.^11^ They found that the prevalence of delirium on admission was significantly lower in palliative home care than in inpatient hospice. They also found that the place of care was not significantly associated with delirium on admission, although they did not adjust for prognostic factors (e.g., Prognosis in Palliative Care Study predictor models) or physical risk factors (e.g., brain metastasis).

Thus, if there is no difference in the prevalence of agitated delirium between palliative home care and PCUs, home care staff will need to become proficient in dealing with agitated delirium. Furthermore, pharmacotherapy and devices need to be developed to control agitated delirium at home, even if they cannot be used internally.

To the best of our knowledge, no study has compared the prevalence of hyperactive delirium between PCUs and palliative home care at the same timing using the same assessment tool or explored associated factors considering the place of care and prognostic factors.

Therefore, we compared the prevalence and associated factors of hyperactive delirium between advanced cancer patients admitted to PCUs and those in palliative home care in Japan on admission and at 3 days before death.

## 2 METHODS

This was a post hoc exploratory analysis of two multicenter, prospective cohort studies of advanced cancer patients who were receiving palliative care in PCUs or at home to addresses the dying process and end‐of‐life care in terminally ill cancer patients, especially to clarify the symptoms of and medical treatment for advanced cancer patients at the end of life. One study was performed at 23 PCUs between Jan 2017 and Dec 2017,^12^ and other was performed on 45 palliative home care services between July 2017 and Dec 2017 in Japan.

The palliative care specialists in PCUs and the primary care physicians with expertise and experience in palliative care in home care were primarily responsible for each patient evaluated and recorded all measurements on the day of enrollment. The physician followed the patient until death or 6 months after enrollment, and the observation period ended when patients were discharged from PCUs or palliative home care either alive or dead. In general, physicians routinely assessed and recorded symptoms and treatments on a daily basis, but in some cases, they assessed and recorded them retrospectively after the observation period based on medical records and memory.

Both studies were conducted in accordance with the ethical standards of the Declaration of Helsinki and the ethical guidelines for research presented by the Ministry of Health, Labour and Welfare of Japan. The institutional review boards of all participating services approved this study, and main institutional review boards (at PCUs: Seirei Mikatahara General Hospital, for home care: University of Tsukuba) approved the use of existing data for secondary analysis and their combination.

## 3 PATIENTS

Eligible patients were enrolled consecutively when admitted to PCUs or starting palliative home care at the participating facilities. The eligibility criteria for the two studies were the same; (a) 18 years old or older, (b) locally advanced or metastatic cancer (including hematopoietic neoplasms), and (c) admitted to PCUs or started palliative home care at the participating facilities during the study period. Patients admitted to PCUs who were expected to be transferred or discharged within a week were excluded.

## 4 MEASUREMENTS

We used item 9 of the Memorial Delirium Assessment Scale (MDAS#9) to assess the subtypes and severity of delirium at the time of assessment.^1^, ^13^, ^14^, ^15^ Agitated delirium was defined as being present when delirium was diagnosed using the DSM5, and classified into either hyperactive or mixed type using item 9 of the MDAS at the time of assessment, which was a score of 2 (moderate) or 3 (severe). In addition, Nagase et al. defined patients who had a score on MDAS#9 of 2 (moderate) or higher as agitated patients.^14^ The rationale for adopting item 9 of the MDAS as an outcome measurement instead of the total MDAS score was that several studies demonstrated that item 9 can distinguish the severity of agitation, and thus be used as an outcome variable to assess hyperactive delirium in terminally ill cancer patients.^13^, ^14^, ^15^

The physicians coded item 9 with “hyperactive features” or “mixed features” based on the last few hours of observations. We assessed the presence of agitated delirium on admission and at 3 days before death. To adjust for background factors with a potential influence on the prevalence of agitated delirium at the time of assessment, we collected several other data on the day of enrollment based on previous studies and discussion among researchers,^11^, ^13^, ^16^, ^17^, ^18^, ^19^ including age, gender, Eastern Co‐operative Oncology Group Performance Status (ECOG PS), central nervous system metastasis, chemotherapy within a month, use of oxygen therapy, use of any catheter, age‐adjusted Charlson Comorbidity Index (ACCI),^20^, ^21^ pleural effusion, asities, symptom severity defined by the Integrated Palliative Care Outcome Scale (IPOS),^22^, ^23^, ^24^ opioid dosage, usage of antipsychotics, usage of benzodiazepines, data to formulate Prognosis in Palliative Care Study predictor models‐A (PiPS‐A),^25^, ^26^ site of primary cancer, metastatic site, Abbreviated Mental Test judged by the physician, heart rate, anorexia, dysphagia, dyspnea, and weight loss in the previous month. We assessed the symptom severity of pain, shortness of breath, weakness or lack of energy, drowsiness, and sore or dry mouth using IPOS, which was scored as 0 (not at all), 1 (slight), 2 (moderate), 3 (severe), and 4 (overwhelming), and defined the prevalence as any IPOS symptoms specified as 2 (moderate), 3 (severe), or 4 (overwhelming).^22^, ^27^

Similarly, we recorded several other data of symptoms and treatment before death, such as the dosage of opioids, fever, and parenteral hydration at 1 week before death, and symptom severity at 3 days before death.

We also recorded the demographic and clinical characteristics of the participants, including the site of primary cancer, presence of bowel obstruction, and data to assess the Palliative Prognostic Index (PPI),^28^ including the Palliative Performance Scale, oral intake, edema, and dyspnea at rest.

## 5 STATISTICAL ANALYSIS

We analyzed patients with a known date of death. First, we conducted descriptive analyses of the demographic characteristics, and compared the prevalence of hyperactive delirium on admission and at 3 days before death between care settings using the chi‐square test or Fisher's exact test. Subsequently, we performed univariate logistic regression analysis of the prevalence of agitated delirium on admission and at 3 days before death between care settings.

We next considered the impact of missing data. Although, the extent of missing data on admission was less than 1%, some data points before death were missing in more than 10%. Thus, we performed multiple imputation based on the missing data at random and the results for 20 imputations were pooled using normalizing transformations.^29^, ^30^

Subsequently, we carried out multivariate logistic regression analysis to adjust for the patient background, symptoms, and treatment with a potential influence on the prevalence of agitated delirium on admission and at 3 days before death.

As possible factors affecting the prevalence of agitated delirium on admission, we used the following independent variables based on previous studies and discussion among the authors^11^, ^13^, ^16^, ^17^, ^18^, ^19^: place of care, age (≥65 years), gender, presence of central nervous system metastasis, chemotherapy within a month, ECOG PS (≥3 on admission), modified PiPS‐A (months, weeks, days), ACCI, using oxygen therapy, using any catheter, and opioid dosage on admission (oral morphine equivalent: OME ≥60 mg/day). Similarly, as possible factors affecting the prevalence of agitated delirium 3 days before death, we used the following independent variables based on previous studies and discussion among the authors^11^, ^13^, ^16^, ^17^, ^18^, ^19^: place of care, age (≥65 years), gender, presence of central nervous system metastasis, chemotherapy within a month, ECOG PS (≥3 on admission), modified PiPS‐A (months, weeks, days), ACCI, opioid dosage 1 week before death (oral morphine equivalent ≥60 mg/day),^18^ symptoms 3 days before death, fever, and parenteral hydration 1 week before death. We defined the presence of symptoms as any IPOS symptoms specified as moderate, severe, or debilitating based on a previous study.^27^

Significance was accepted at *p* < .05 and analyses were performed using SPSS‐J software (version 25.0; IBM, Tokyo, Japan).

## 6 RESULT

**FIGURE 1:**
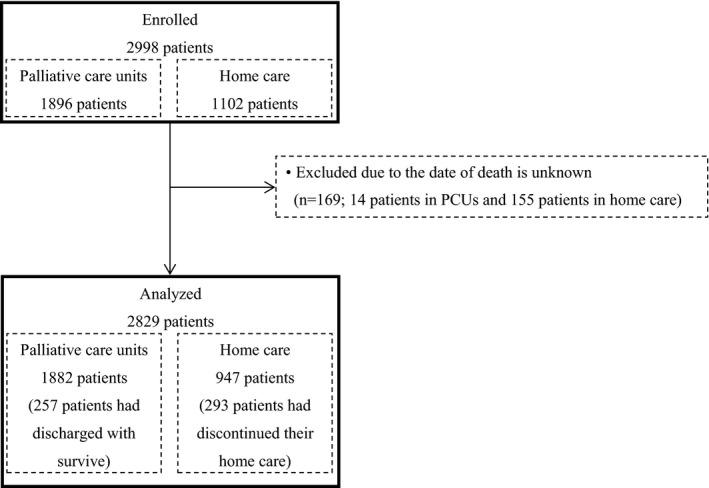
Participants flow

In total, 2998 patients were enrolled in both studies: 1896 patients in PCUs and 1102 patients in palliative home care. Among them, 169 patients were excluded due to an unknown date of death; 14 patients in PCUs and 155 patients in palliative home care. The remaining 2829 patients were analyzed. Two hundred and fifty‐seven patients in PCUs who were discharged survived and 293 patients in palliative home care discontinued their home care. (Figure 1).

The patient characteristics at enrollment are shown in Table 1. The mean age of the subjects was 72.4 ± 12.2 years. Almost 10% of the patients had metastasis to the central nervous system and approximately one‐third had a daily prognosis predicted by the modified PiPS‐A. The prevalence of patients who had delirium and/or agitated delirium on admission was shown in Appendix 1. A total of 672 patients (23.8%) had delirium on admission and 900 (31.8%) had hyperactive or mixed‐type delirium during the study period. A total of 1690 patients (59.7%) were on opioids on admission and the mean opioid dosage was 39.9 mg/day (range: 0–1680 mg/day).

**TABLE 1:** Patient characteristics on enrollment

	All patients (n = 2829)		PCU (n = 1882)		Home care (n = 947)		*p* value
	N	%	N	%	N	%	
Age≥65	2209	78.1	1450	77.0	759	80.1	0.061
Male sex	1492	52.7	959	51.0	533	56.3	0.008
Married	1824	64.5	1147	60.9	677	71.5	< 0.001
Live with family	2192	77.5	1368	72.7	824	87.0	< 0.001
Underage child	117	4.1	74	3.9	43	4.5	0.485
Site of primary cancer							0.084
Gastrointestinal	806	28.5	522	27.7	284	30.0	
Hepatobiliary and pancreas	557	19.7	362	19.2	195	20.6	
Lung	485	17.1	315	16.7	70	7.4	
Urogenital	213	7.5	140	7.4	73	7.7	
Breast	183	6.5	131	7.0	52	5.5	
Gynecological	176	6.2	118	6.3	58	6.1	
Others	409	14.5	294	15.6	115	12.1	
Metastatic site							
Anywhere	2295	81.1	1599	85.0	696	73.5	< 0.001
Liver	1059	37.4	727	38.6	332	35.1	0.077
Bone	702	24.8	498	26.5	204	21.5	0.005
Lung	966	34.1	704	37.4	262	27.7	< 0.001
Central nervous system	367	13.0	262	13.9	105	11.1	0.038
Chemotherapy within a month	360	12.7	170	9.0	190	20.1	< 0.001
ECOG PS^a^							< 0.001
0–1	122	4.3	24	1.3	98	10.3	
2	358	12.7	155	8.2	203	21.4	
3	1153	40.8	790	42.0	363	38.3	
4	1196	42.3	913	48.5	283	29.9	
modified PiPs‐A^b^							< 0.001
Months	482	17.0	246	13.1	236	24.9	
Weeks	1409	49.8	891	47.3	518	54.7	
Days	907	32.1	721	38.3	186	19.6	
Palliative Prognostic Index6.5	903	31.9	737	39.2	166	17.5	< 0.001
Oxygen therapy	699	24.7	566	30.1	133	14.0	< 0.001
Use of any catheter	538	19.0	452	24.0	86	9.1	< 0.001
Delirium (DSM‐Ⅴ) on admission	672	23.8	580	30.8	92	9.7	< 0.001
Pain IPOS^c^ ≥2	1013	35.8	663	35.2	350	37.0	0.933
Shortness of breath IPOS ≥2	583	20.6	379	20.1	204	21.5	0.768
Weakness or lack of energy IPOS ≥2	1192	42.1	785	41.7	407	43.0	0.714
Drowsiness IPOS ≥2	592	20.9	413	21.9	179	18.9	0.013
Sore or dry mouth IPOS ≥2	497	17.6	361	19.2	136	14.4	< 0.001
Anorexia	2338	82.6	1546	82.1	792	83.6	0.371
Dysphagia	820	29.0	622	33.0	198	20.9	< 0.001
Weight loss in the previous month	2130	75.3	1376	73.1	754	79.6	< 0.001
Edema	1237	43.7	867	46.1	370	39.1	< 0.001
Pleural effusion	744	26.3	552	29.3	192	20.3	< 0.001
Ascites	846	29.9	567	30.1	279	29.5	0.728
Bowel obstruction	353	12.5	256	13.6	97	10.2	0.011
Use of opioid	1690	59.7	1197	63.6	493	52.1	< 0.001

	Mean	95% CI	Mean	95% CI	Mean	95% CI	
Age (yrs)	72.4	72.0 – 72.9	72.3	71.8 – 72.9	72.7	71.9 – 73.4	0.460
Age‐adjusted Charlson comorbidity index	10.8	10.7 – 10.9	11.0	10.9 – 11.1	10.5	10.3 – 10.6	< 0.001
Palliative Prognostic Index	5.4	5.3 – 5.6	6.1	5.9 – 6.2	4.2	4.0 – 4.4	< 0.001
Opioid dosage per day (OME^d^, mg/day)	39.9	36.7 – 43.1	43.5	39.6 – 47.4	32.8	27.2 – 38.4	0.002
Survival (days)	31.8	30.6 – 32.9	26.0	24.8 – 27.2	43.2	40.7 – 45.7	< 0.001

^a^ ECOG PS: Eastern Co‐operative Oncology Group Performance Status.

^b^ PiPs‐A: Prognosis in Palliative Care Study predictor models‐A.

^c^ IPOS: Integrated Palliative Care Outcome Scale.

^d^ OME: Oral morphine equivalent.

The prevalence of symptoms and treatment at 1 week and 3 days before death after multiple imputation is shown in Appendix 2, –4.

### 6.1 Comparison of the prevalence of agitated delirium on admission and at 3 days before death without adjusting for the patient background

The prevalence of agitated delirium on admission was 3.9% (95% CI: 3.2–4.7) among all patients, and there was significant difference between PCUs and palliative home care; 5.2% (95% CI: 4.2%–6.3%) vs. 1.4% (0.7%–2.3%) (*p* < 0.001). (Table 2) The prevalence of agitated delirium 3 days before death after multiple imputation was 6.9% (95% CI: 6.0 – 7.9) among all patients, and there was a significant difference between PCUs and palliative home care; 7.6% (6.4% –8.9%) vs. 5.4% (4.0% –7.0%) (*p* < 0.001). (Table 2).

**TABLE 2:** Prevalence of hyperactive delirium

	All patients (n = 2829)		PCUs (n = 1882)		Home care (n = 947)		
	N	%	N	%	N	%	*p*‐value
On admission	110	3.9	97	5.2	13	1.4	< 0.001
3 days before death	194	6.9	143	7.6	51	5.4	< 0.001

Hyperactive delirium defined by item 9 of the Memorial Delirium Assessment Scale (MDAS#9) was a score of 2 (moderate) or 3 (severe). The number of hyperactive delirium patients is the actual number of patients not corrected by multiple imputation.

### 6.2 Multivariate logistic regression analysis of the prevalence of agitated delirium on admission and at 3 days before death

Multivariate logistic regression analysis revealed that home care was significantly associated with the lower prevalence of agitated delirium on admission (OR: 0.42, *p* = 0.006), but it was not significantly associated with the prevalence of hyperactive features at 3 days before death (OR: 0.74, *p* = 0.173). (Tables 3 and 4) OR: 1.85, *p* = 0.011, OR: 4.43, *p* < 0.001), Male gender, central nervous system metastasis, and use of any catheter were significantly associated with the higher prevalence of agitated delirium on admission (OR: 2.42, *p* < 0.001, OR: 1.94, *p* = 0.015, OR: 1.84, *p* = 0.008). (Table 3) At 3 days before death, male gender and weakness or lack of energy were significantly positively associated with the prevalence of agitated delirium (OR: 1.50, *p* = 0.033, OR: 2.39, *p* < 0.001). (Table 4) The use of opioids was not significantly associated with the prevalence of agitated delirium on admission or at 3 days before death.

**TABLE 3:** Multivariate logistic regression analysis for factors associated with the prevalence of hyperactive delirium on admission

	OR	95% CI	*p*
Unadjusted model: home care	0.26	0.14– 0.46	<0.001
Adjusted model			
Home care	0.42	0.23– 0.78	0.006
65 years	0.99	0.59– 1.66	0.960
Male	2.42	1.54– 3.83	<0.001
Central nervous system metastasis	1.94	1.14– 3.31	0.015
Chemotherapy within a month	0.92	0.46– 1.84	0.810
ECOG PS^a^≥3 at enrollment	2.42	0.71– 8.23	0.158
modified PiPs‐A^b^			
Months	n.a	n.a	n.a
Weeks	1.49	0.56– 4.00	0.426
Days	3.85	1.43– 10.38	0.008
Age‐adjusted Charlson comorbidity index	0.98	0.89– 1.07	0.618
Symptom and treatment on admission			
Use of any catheter	1.84	1.18– 2.87	0.008
Pain IPOS^c^ ≥2	1.38	0.89– 2.13	0.146
Shortness of breath IPOS ≥2	1.12	0.69– 1.81	0.640
Weakness or lack of energy IPOS ≥2	1.17	0.74– 1.86	0.494
Drowsiness IPOS ≥2	1.25	0.77– 2.03	0.371
Sore or dry mouth IPOS ≥2	1.07	0.65– 1.78	0.792
Ascites	0.83	0.51– 1.37	0.468
Using opioid	1.50	0.91– 2.48	0.116

We conducted the multivariate logistic regression analysis using categorical variables, except for the age‐adjusted Charlson Comorbidity Index. The number for each factor represents the actual number of patients not corrected by multiple imputation.

^a^ ECOG PS: Eastern Co‐operative Oncology Group Performance Status.

^b^ PiPs‐A: Prognosis in Palliative Care Study predictor models‐A.

^c^ IPOS: Integrated Palliative Care Outcome Scale.

**TABLE 4:** Multivariate logistic regression analysis for factors associated with the prevalence of hyperactive delirium 3 days before death

	OR	95% CI	*p*
Unadjusted model: home care	0.87	0.62– 1.23	0.436
Adjusted model			
Home care	0.74	0.48– 1.14	0.173
65 years	0.79	0.52– 1.20	0.265
Male	1.50	1.03– 2.17	0.033
Central nervous system metastasis	0.86	0.46– 1.60	0.638
Chemotherapy within a month	1.05	0.64– 1.73	0.854
modified PiPs‐A^a^			
Months	n.a	n.a	n.a
Weeks	0.81	0.47– 1.38	0.433
Days	1.18	0.67– 2.08	0.565
Age‐adjusted Charlson comorbidity index	1.05	0.97– 1.14	0.194
Using opioid at 1 week before death	2.07	0.97– 4.43	0.061
Symptom and treatment 3 days prior to death			
Weakness or lack of energy IPOS^b^ ≥2	2.39	1.52– 3.74	<0.001
Drowsiness IPOS ≥2	0.78	0.51– 1.18	0.240
Sore or dry mouth IPOS ≥2	1.46	0.98– 2.19	0.064
Shortness of breath at rest	1.04	0.71– 1.51	0.859
Ascites	0.69	0.41– 1.16	0.158

We conducted the multivariate logistic regression analysis using categorical variables, except for the age‐adjusted Charlson Comorbidity Index. The number for each factor was corrected by multiple imputation.

^a^ PiPs‐A: Prognosis in Palliative Care Study predictor models‐A.

^b^ IPOS: Integrated Palliative Care Outcome Scale.

## 7 DISCUSSION

To the best of our knowledge, this is the first large‐scale post hoc exploratory analysis of two multicenter, prospective cohort studies to compare the prevalence and possible associated factors of agitated delirium on admission and at 3 days before death between advanced cancer patients in PCUs and those in palliative home care.

The most important findings of our study were that there was no significant difference in the prevalence of agitated delirium at 3 days before death between PCUs and palliative home care after adjusting for the patient background, prognostic factors, symptoms, and treatment, although there was significant difference on admission by the unadjusted analysis.

Our study suggested that the place of care is not associated with the prevalence of agitated delirium before death, although the prevalence on admission was significantly lower in home care settings. A recent systematic review revealed that the rate of delirium at the end of life was 78%‐85% in inpatient settings, whereas it was 42.5%‐44% in the community setting, though the timing and assessment tools were not standardized.^10^ Watt et al. considered this to be possibly related to home care being less disorienting and the condition of patients in home care being less complex.^10^ However, our study suggested that the home care setting was not associated with the prevalence of agitated delirium at 3 days before death, although it was associated on admission.

The second important finding of this study was that opioid usage was not significantly associated with the prevalence of agitated delirium in terminally ill cancer patients. However, Senel et al. reported that the use of opioids (used or not used) was one of the risk factors for delirium among cancer patients in PCUs.^17^ Lim et al. also found that a high daily morphine equivalent dose at the start of palliative care consultation was positively associated with the incidence of opioid‐induced neurotoxicity, which includes delirium.^18^ One possible reason for the different results is that we may have underestimated the prevalence of agitated delirium because our assessment was based on point estimation.

Of note, although a recent systematic review revealed the prevalence of hyperactive delirium in the palliative care setting to be 14% (0%‐33%),^10^ the prevalence of agitated delirium in our study was 3.9% on admission and 6.9% at 3 days before death. One possible reason for the lower prevalence of agitated delirium than in the previous study was that we were unable to assess the presence of hyperactive delirium throughout the observation periods as we described in limitations. Another possible reason was the underestimation of mixed‐type delirium, as noted by de la Cruz et al.^3^ In addition, as hypoactive delirium is difficult to detect but could affect the quality of death and dying,^1^, ^2^, ^3^, ^31^ future study should include the prevalence and impact of hypoactive delirium on patients and families as well as those of hyperactive/mixed type of delirium.

Our results suggest that health care providers, especially in the home care setting, need to become proficient in dealing with agitated delirium before death. Furthermore, pharmacotherapy and devices need to be developed to control agitated delirium at home, even if they cannot be used internally.

In terms of the direction for further studies to clarify the prevalence of hyperactive delirium in the palliative care setting, daily universal screening for delirium and analysis using a variety of adjustment factors is warranted.

The current study had several limitations. First, we defined agitated delirium as being present when delirium was diagnosed using the DSM5 and classified into either the hyperactive or mixed type using item 9 of the MDAS on admission and 3 days before death. Therefore, we were unable to assess the presence of hyperactive delirium throughout the observation periods. In addition, we were unable to assess mixed‐type delirium patients who exhibited hypoactive features at the time of assessment. These reasons may explain the low prevalence of agitated delirium in our study. Second, we were unable to adjust for residual confounding factors affecting the development of agitated delirium at the time of assessment (e.g. use of corticosteroids and degree of cognitive impairment). The possibility that these factors affected the development of agitated delirium at the time of assessment cannot be excluded. For this reason, we were unable to conclude the definitive factors of agitated delirium at the time of assessment in advanced cancer patients in PCUs or palliative home care.

Third, the symptoms and treatment during death were not always evaluated prospectively. Therefore, exploration of the potential effects of symptoms and treatment during death on the prevalence of agitated delirium at the time of assessment is required.

Fourth, approximately one‐third of the palliative home care patients discontinued their home care, and most were hospitalized and died. The reasons for hospitalization were not assessed. Therefore, we were unable to assess the rate of home care patients admitted to the hospital due to agitated delirium at the time of assessment. However, we performed multiple imputation to minimize this limitation. Thus, the prevalence of agitated delirium at the time of assessment in palliative home care may be higher than reported. Future studies are needed to assess daily symptoms and clarify the prevalence of hyperactive delirium in palliative home care.

Fifth, we defined agitated delirium at the time of assessment only based on item 9 of the MDAS, which has not been validated by the total MDAS score. A recent review noted MDAS as one of the best diagnostic tools^32^ and several previous studies diagnosed hyperactive delirium using the total MDAS score ≥7.^3^, ^11^, ^16^ However, several studies demonstrated that item 9 is associated with the neurobehavioral dimension and severity of agitation, and they used it as an outcome variable to assess hyperactive delirium in terminally ill cancer patients.^13^, ^14^, ^15^ Although this limitation was unlikely to have a significant impact on the results of our study, the validity and reliability need to be assessed between the total MDAS score and item 9 of the MDAS.

## 8 CONCLUSION

There was no significant difference in the prevalence of agitated delirium at 3 days before death between PCUs and palliative home care after adjusting for the patient background, prognostic factors, symptoms, and treatment.

## 9 FUNDING SOURCE

This EASED study was supported in part by a Grant‐in‐Aid from the Japanese Hospice Palliative Care Foundation. The sponsor played no role in study design, collection, analysis, or interpretation of the data, writing of the report, or in the decision to submit the paper for publication.

This study was performed in the Comparison of End‐of‐life trajectory in advanced cancer patient between inpatient hospice and home (Come Home study) and the East‐Asian collaborative cross‐cultural Study to Elucidate the Dying process (EASED).

The participating investigators and study sites of Come Home study were as follows: Keijiro Miyake, M.D., Ph.D. (Keijiro Clinic), Manabu Tamura, M.D., Ph.D. (Osaka Home Healthcare Clinic), Junichiro Toya, M.D. (Sakura‐shinmachi Urban Clinic), Hiroto Shirayama, M.D. (Osaka Kita Home Care Clinic), Takamichi Matsuki, M.D., Ph.D. (Fujisawa‐Honmachi Family Clinic), Akihiro Ishikawa, M.D., (Ishikawa Rehabili Noushinkeigeka Clinic), Yasunori Muraoka, M.D. (Muraoka Home Clinic), Yasuhiro Saitou, M.D. (GP Clinic Jiyugaoka), Takahide Yamaguchi, M.D. (Yamaguchi Clinic), Tomohiro Nishi, M.D. (Kawasaki Municipal Ida Hospital), Nobuyuki Miyata, M.D. (Miyata Clinic), Masakatsu Shimizu, M.D., Ph.D. (Shimizu Medical Clinic), Ryo Yamamoto, M.D. (Saku Central Hospital Advanced Care Center), Yousuke Kimura, M.D. (Yamato Clinic), Yoshiyuki Kamiyama, M.D. (Okinawa Chubu Hospital), Yasuyuki Arai, M.D., Ph.D. (Iki‐iki Clinic), Daishi Matsuo, M.D. (Margaret Clinic), Hideki Shishido, M.D. (Shishido Internal Medicine Clinic), Kazushi Nakano, M.D., Ph.D. (Nakano Zaitakuiryou Clinic), Kan Asahina, M.D. (Mutsumimachi Clinic), Maiko Haruki, M.D. (Orange Home‐Care Clinic), Keiko inoue, M.D. (Aisei clinic), Sen Otomo, M.D., (Seimeikan Clinic).

The participating investigators and study sites of EASED were as follows: Satoshi Inoue, M.D. (Seirei Hospice, Seirei Mikatahara General Hospital), Naosuke Yokomichi, M.D., Ph.D. (Department of Palliative and Supportive Care, Seirei Mikatahara General Hospital), Hiroaki Tsukuura, M.D., Ph.D. (Department of Palliative Care, TUMS Urayasu Hospital), Toshihiro Yamauchi, M.D. (Seirei Hospice, Seirei Mikatahara General Hospital), Akemi Shirado Naito, M.D. (Department of palliative care Miyazaki Medical Association Hospital), Yu Uneno, M.D. (Department of Therapeutic Oncology, Graduate School of Medicine, Kyoto University), Akira Yoshioka, M.D., Ph.D. (Department of Oncology and Palliative Medicine, Mitsubishi Kyoto Hospital), Shuji Hiramoto, M.D. (Department of Oncology and Palliative Medicine, Mitsubishi Kyoto Hospital), Ayako Kikuchi, M.D. (Department of Oncology and Palliative Medicine, Mitsubishi Kyoto Hospital), Tetsuo Hori, M.D. (Department of Respiratory surgery, Mitsubishi Kyoto Hospital), Yosuke Matsuda, M.D. (Palliative Care Department, St.Luke's International Hospital), Hiroyuki Kohara, M.D., Ph.D. (Hiroshima Prefectural Hospital), Hiromi Funaki, M.D. (Hiroshima Prefectural Hospital), Keiko Tanaka, M.D., Ph.D. (Department of Palliative Care Tokyo Metropolitan Cancer & Infectious Diseases Center Komagome Hospital), Kozue Suzuki, M.D. (Department of Palliative Care Tokyo Metropolitan Cancer & Infectious Diseases Center Komagome Hospital), Tina Kamei, M.D. (Department of Palliative Care, NTT Medical Center Tokyo), Koji Amano, M.D. (Department of Palliative Medicine, Osaka City General Hospital), Teruaki Uno, M.D. (Department of Palliative Medicine, Osaka City General Hospital), Jiro Miyamoto, M.D. (Department of Palliative Medicine, Osaka City General Hospital), Hirofumi Katayama, M.D. (Department of Palliative Medicine, Osaka City General Hospital), Hideyuki Kashiwagi, M.D., MBA. (Aso Iizuka Hospital / Transitional and Palliative Care), Eri Matsumoto, M.D. (Aso Iizuka Hospital / Transitional and Palliative Care), Takeya Yamaguchi, M.D. (Japan Community Health care Organization Kyushu Hospital / Palliative Care), Tomonao Okamura, M.D., MBA. (Aso Iizuka Hospital / Transitional and Palliative Care), Hoshu Hashimoto, M.D., MBA. (Inoue Hospital / Internal Medicine), Shunsuke Kosugi, M.D. (Department of General Internal Medicine, Aso Iizuka Hospital), Nao Ikuta, M.D. (Department of Emergency Medicine, Osaka Red Cross Hospital), Yaichiro Matsumoto, M.D. (Department of Transitional and Palliative Care, Aso Iizuka Hospital), Takashi Ohmori, M.D. (Department of Transitional and Palliative Care, Aso Iizuka Hospital), Takehiro Nakai, M.D. (Immuno‐Rheumatology Center, St Luke's International Hospital), Takashi Ikee, M.D. (Department of Cardiorogy, Aso Iizuka Hospital), Yuto Unoki, M.D. (Department of General Internal Medicine, Aso Iizuka Hospital), Kazuki Kitade, M.D. (Department of Orthopedic Surgery, Saga‐Ken Medical Centre Koseikan), Shu Koito, M.D. (Department of General Internal Medicine, Aso Iizuka Hospital), Nanao Ishibashi, M.D. (Environmental Health and Safety Division, Environmental Health Department, Ministry of the Environment), Masaya Ehara, M.D. (TOSHIBA), Kosuke Kuwahara, M.D. (Department of General Internal Medicine, Aso Iizuka Hospital), Shohei Ueno, M.D. (Department of Hematology / Oncology, Japan Community Healthcare Organization Kyushu Hospital), Shunsuke Nakashima, M.D. (Oshima Clinic), Yuta Ishiyama, M.D. (Department of Transitional and Palliative Care, Aso Iizuka Hospital), Akihiro Sakashita, M.D., Ph.D. (Department of Palliative Medicine, Kobe University School of Medicine), Hana Takatsu, M.D. (Division of Palliative Care, Konan Medical Center), Takashi Yamaguchi, M.D., Ph.D. (Division of Palliative Care, Konan Medical Center), Satoko Ito, M.D. (Hospice, The Japan Baptist Hospital), Toru Terabayashi, M.D. (Hospice, The Japan Baptist Hospital), Jun Nakagawa, M.D. (Hospice, The Japan Baptist Hospital), Tetsuya Yamagiwa, M.D., Ph.D. (Hospice, The Japan Baptist Hospital), Akira Inoue, M.D., Ph.D. (Department of Palliative Medicine Tohoku University School of Medicine), Takuhiro Yamaguchi, Ph.D. (Professor of Biostatistics, Tohoku University Graduate School of Medicine), Mitsunori Miyashita, R.N., Ph.D. (Department of Palliative Nursing, Health Sciences, Tohoku University Graduate School of Medicine), Saran Yoshida, Ph.D. (Graduate School of Education, Tohoku University), Yusuke Hiratsuka, M.D., Ph.D. (Department of Palliative Medicine Tohoku University School of Medicine), Keita Tagami, M.D., Ph.D. (Department of Palliative Medicine Tohoku University School of Medicine), Hiroaki Watanabe, M.D. (Department of Palliative Care, Komaki City Hospital), Takuya Odagiri, M.D. (Department of Palliative Care, Komaki City Hospital), Tetsuya Ito, M.D.,Ph.D. (Department of Palliative Care, Japanese Red Cross Medical Center), Masayuki Ikenaga, M.D. (Hospice, Yodogawa Christian Hospital), Keiji Shimizu, M.D., Ph.D. (Department of Palliative Care Internal Medicine, Osaka General Hospital of West Japan Railway Company), Akira Hayakawa, M.D., Ph.D. (Hospice, Yodogawa Christian Hospital), Rena Kamura, M.D. (Hospice, Yodogawa Christian Hospital), Takeru Okoshi, M.D., Ph.D. (Okoshi Nagominomori Clinic), Isseki Maeda M.D., Ph.D. (Department of Palliative Care, Senri‐Chuo Hospital), Tomohiro Nishi, M.D. (Kawasaki Municipal Ida Hospital, Kawasaki Comprehensive Care Center), Kazuhiro Kosugi, M.D. (Department of Palliative Medicine, National Cancer Center Hospital East), Yasuhiro Shibata, M.D. (Kawasaki Municipal Ida Hospital, Kawasaki Comprehensive Care Center), Takayuki Hisanaga, M.D. (Department of Palliative Medicine, Tsukuba Medical Center Hospital), Takahiro Higashibata, M.D., Ph.D. (Department of General Medicine and Primary Care, Palliative Care Team, University of Tsukuba Hospital), Ritsuko Yabuki, M.D. (Department of Palliative Medicine, Tsukuba Medical Center Hospital), Shingo Hagiwara, M.D., Ph.D. (Department of Palliative Medicine, Yuai Memorial Hospital), Miho Shimokawa, M.D. (Department of Palliative Medicine, Tsukuba Medical Center Hospital), Satoshi Miyake, M.D., Ph.D. (Professor, Department of Clinical Oncology Graduate School of Medical and Dental Sciences Tokyo Medical and Dental University (TMDU)), Junko Nozato, M.D. (Specially Appointed Assistant Professor, Department of Internal Medicine, Palliative Care, Medical Hospital, Tokyo Medical and Dental University), Hiroto Ishiki, M.D. (Department of Palliative Medicine, National Cancer Center Hospital), Tetsuji Iriyama, M.D. (Specially Appointed Assistant Professor, Department of Internal Medicine, Palliative Care, Medical Hospital, Tokyo Medical and Dental University), Keisuke Kaneishi, M.D., Ph.D. (Department of Palliative Care Unit, JCHO Tokyo Shinjuku Medical Center), Tomofumi Miura, M.D., Ph.D. (Department of Palliative Medicine, National Cancer Center Hospital East), Yoshihisa Matsumoto, M.D., Ph.D. (Department of Palliative Medicine, National Cancer Center Hospital East), Ayumi Okizaki, Ph.D. (Department of Palliative Medicine, National Cancer Center Hospital East), Yuki Sumazaki Watanabe, M.D. (Department of Palliative Medicine, National Cancer Center Hospital East), Yuko uehara, M.D. (Department of Palliative Medicine, National Cancer Center Hospital East), Eriko Satomi, M.D. (Department of palliative medicine, National Cancer Center Hospital), Kaoru Nishijima, M.D. (Department of Palliative Medicine, Kobe University Graduate School of Medicine), Junichi Shimoinaba, M.D. (Department of Hospice Palliative Care, Eikoh Hospital), Ryoichi Nakahori, M.D. (Department of Palliative Care, Fukuoka Minato Home Medical Care Clinic), Takeshi Hirohashi, M.D. (Eiju General Hospital), Jun Hamano, M.D., Ph.D. (Assistant Professor, Faculty of Medicine, University of Tsukuba), Natsuki Kawashima, M.D. (Department of Palliative Medicine, Tsukuba Medical Center Hospital), Takashi Kawaguchi, Ph.D. (Tokyo University of Pharmacy and Life Sciences Department of Practical Pharmacy), Megumi Uchida, M.D., Ph.D. (Dept. of Psychiatry and Cognitive‐Behavioral Medicine, Nagoya City University Graduate School of Medical Sciences), Ko Sato, M.D., Ph.D. (Hospice, Ise Municipal General Hospital), Yoichi Matsuda, M.D., Ph.D. (Department of Anesthesiology & Intensive Care Medicine / Osaka University Graduate School of Medicine), Yutaka Hatano, M.D., Ph.D. (Hospice, Gratia Hospital), Satoru Tsuneto, M.D., Ph.D. (Professor, Department of Human Health Sciences, Graduate School of Medicine, Kyoto University Department of Palliative Medicine, Kyoto University Hospital), Sayaka Maeda, M.D. (Department of Palliative Medicine, Kyoto University Hospital), Yoshiyuki Kizawa M.D., Ph.D., FJSIM, DSBPMJ. (Designated Professor and Chair, Department of Palliative Medicine, Kobe University School of Medicine), Hiroyuki Otani, M.D. (Palliative Care Team, and Palliative and Supportive Care, National Kyushu Cancer Center).

## CONFLICT OF INTEREST

None of the authors have any financial or personal relationships to declare.

## Data Availability

The datasets used and/or analyzed during the current study are not available.

## APPENDIX A 1. The prevalence of patients who had delirium and/or agitated delirium on admission

**Table:**

	Agitated delirium (MDAS item 9) on admission								
		No episode of agitated or hypoactive delirium		Mild		Moderate		Severe	
		N	%	N	%	N	%	N	%
Delirium (DSM‐Ⅴ) on admission	Yes (n = 672)	318	47.3	244	36.3	93	13.8	17	2.5
	No (n = 2155)	2146	99.6	9	0.4	0	0.0	0	0.0

## APPENDIX A 2. Prevalence of symptoms and treatment at 1 week and 3 days before death in all patients after multiple imputation

**Table:**

	After multiple imputation (n = 2501)	
	N	%
3 days before death		
Hyperactive delirium	211	8.4
Weakness or lack of energy IPOS ≥2	1442	57.7
Drowsiness IPOS ≥2	1031	41.2
Sore or dry mouth IPOS ≥2	808	32.3
Shortness of breath at rest	664	26.5
Ascites	372	14.9
1 week before death		
Fever	800	32.0
Parenteral hydration	1089	43.5
Opioid dosage (OME ≥60 mg/day)	793	31.7

IPOS: Integrated Palliative Care Outcome Scale.

OME: Oral morphine equivalent.

## APPENDIX A 3. Prevalence of symptoms and treatment at 1 week and 3 days before death of patients in palliative care units

**Table:**

	After multiple imputation (n = 1625)	
	N	%
3 days before death		
Hyperactive delirium	143	8.8
Weakness or lack of energy IPOS ≥2	953	58.6
Drowsiness IPOS ≥2	664	40.9
Sore or dry mouth IPOS ≥2	541	33.3
Shortness of breath at rest	462	28.4
Ascites	242	14.9
1 week before death		
Fever	615	37.8
Parenteral hydration	905	55.7
Opioid dosage (OME ≥60 mg/day)	625	38.5

## APPENDIX A 4. Prevalence of symptoms and treatment at 1 week and 3 days before death in patients in home care

**Table:**

	After multiple imputation (n = 876)	
	N	%
3 days before death		
Hyperactive delirium	68	7.8
Weakness or lack of energy IPOS ≥2	489	55.8
Drowsiness IPOS ≥2	367	41.9
Sore or dry mouth IPOS ≥2	266	30.4
Shortness of breath at rest	202	23.1
Ascites	130	14.8
1 week before death		
Fever	185	21.1
Parenteral hydration	185	21.1
Opioid dosage (OME ≥60 mg/day)	182	20.8
